# Cross-lagged relationship between home numeracy practices and early mathematical skills among Chinese young children

**DOI:** 10.3389/fpsyg.2022.1033065

**Published:** 2022-12-07

**Authors:** Wei Wei, Qi-Yi Wang, Qin Luo, Yan Li

**Affiliations:** ^1^Early Childhood Education College, Shanghai Normal University, Shanghai, China; ^2^Early Child Development Research Center, Shanghai Normal University, Shanghai, China

**Keywords:** home numeracy practices, early mathematical skills, cross-lagged, basic number processing, arithmetic skills

## Abstract

The present study examined the cross-lagged relationship between home numeracy practices (e.g., formal teaching, number games, and number application) and early mathematical skills (basic number processing, and arithmetic skills) among Chinese young children. A total of 155 children (82 boys; mean age = 67.49 months, SD = 3.58 months) were assessed with basic number processing and arithmetic skills at three timepoints during the kindergarten year, and their parents reported the frequency of parent–child numeracy activities. Main results from random-intercept cross-lagged panel models showed that, at the within-family level, earlier basic teaching activities uniquely predicted subsequent basic number processing, while both advanced teaching activities and number game activities at earlier timepoints predicted the following arithmetic skills. These results indicated a unidirectional effect from home numeracy practices on early mathematical skills during the early years.

## Introduction

The past decade of studies showed that children’s learning experience with parents on numeracy, i.e., home numeracy practices, were associated with their mathematics skills (e.g., Huntsinger et al., 2000; Lefevre et al., 2002, 2009, 2010; Kleemans et al., 2012; Manolitsis et al., 2013; Niklas and Schneider, 2014; Skwarchuk et al., 2014; Deng et al., 2015; Huang et al., 2017; Susperreguy et al., 2020; Wei et al., 2020). Most of these studies examined the concurrent relations between home numeracy practices and mathematics skills, and the researchers assumed that home numeracy practices unidirectionally predicted mathematical skills. However, far less was known about the cross-lagged relationship between home numeracy practices and early mathematical skills. Examining their mutual relations may help understand not only the specific role of parent–child numeracy activities in children’s early mathematics learning, but also the influence of children’s mathematics performance on parental involvement in numeracy activities. Thus, the present study aimed to examine the cross-lagged relationship between different aspects of home numeracy practices (formal teaching, number games, and number application) and early mathematical skills (basic number processing, arithmetic skills) during the kindergarten year.

Previous studies showed that parents may involve in a wide range of mathematics-related activities (Lefevre et al., 2002; Deng et al., 2015), such as teaching their child number knowledge directly, playing number games with their child, and using the number in everyday life. In several studies, home numeracy practices are typically composed of formal and informal numeracy activities based on whether parents use an explicit or implicit way (e.g., Lefevre et al., 2010; Skwarchuk et al., 2014; Deng et al., 2015). In formal numeracy activities, parents directly provide instructions on number knowledge and arithmetic procedure. While in informal numeracy activities, parents engage their children in games related to numbers (such as board games with dice) or talking about numbers in everyday life (such as prices during shopping).

The relationship between home numeracy practices and mathematical skills has been well established in the past decade (e.g., Lefevre et al., 2002, 2010; Silinskas et al., 2010, 2020; Kleemans et al., 2012; Niklas and Schneider, 2014; Skwarchuk et al., 2014; Deng et al., 2015; Huang et al., 2017; Mutaf-Yıldız et al., 2020, for a review; Wei et al., 2020). A recent meta-analysis study by Daucourt et al. (2021) indicated an average correlation of 0.13 between home mathematics environment and children’s mathematics. Furthermore, their results showed that the relation between home numeracy practices and mathematical skills during the early year was much higher than that during formal schooling.

In the review of Mutaf Yıldız et al. (2018), most studies with young children examined mathematic skills with comprehensive tests on a group of numeracy knowledge and arithmetic skills, while far fewer studies examined how two types of home numeracy practices were related to specific mathematics skills (e.g., basic number processing, arithmetic skills). Results of these studies (Mutaf Yıldız et al., 2018; Vasilyeva et al., 2018; Susperreguy et al., 2020) showed that numeracy activities were differently related to basic number processing and arithmetic skills. More specifically, both formal and informal numeracy practices were uniquely related to arithmetic skills in most of these studies (e.g., LeFevre et al., 2009; Dearing et al., 2012; Huang et al., 2017; Mutaf Yıldız et al., 2018, for exception; Vasilyeva et al., 2018; Susperreguy et al., 2020), while children’s number processing was explained by formal numeracy activities in one study (Susperreguy et al., 2020) but informal numeracy activities in another (Vasilyeva et al., 2018). For example, Susperreguy et al. (2020) found formal numeracy activities (including manipulation of digits or quantities) in prekindergarten years uniquely predicted number processing (non-symbolic and symbolic comparison) 1 year later, while both formal numeracy activities and shared-number games play uniquely predicted arithmetic skills 1 year later.

When explaining their relationship, most researchers (e.g., Skwarchuk et al., 2014; Susperreguy et al., 2020), from the sociocultural learning theory by Vygotsky (1978), claimed that children develop their early mathematical skills through the interactions during parent–child numeracy activities. However, children’s mathematical skills may also have influence on parents’ activities. According to Rutter (1997), children’s characteristics and behaviors may elicit parents’ particular responses. To date, three longitudinal studies (e.g., Silinskas et al., 2010, 2020; Deng et al., 2015) found that primary students’ earlier performance on mathematics may also predict later home numeracy practices, but negatively. Deng et al. (2015) argued that parents of primary students would give more frequent numeracy practices when they learned about children’s poor performance in school from teachers (e.g., the test reports), which, in one recent study by Silinskas et al. (2020), was referred to be *responsive home numeracy* to children’s mathematics performance.

Therefore, not only the effects of home numeracy practices on mathematics but also the reverse effects should be examined in longitudinal studies. However, to our knowledge, only three studies (Silinskas et al., 2010, 2020; Deng et al., 2015) had examined their bidirectional relationships, and all three studies examined primary students. Parents of primary students may learn their children’s mathematics performance through homework and the feedback from school (e.g., test reports), while parents of kindergarten children may have less ways to know children’s mathematics skills due to no test reports and much less homework in kindergarten (Pressman et al., 2015). Therefore, it is unknown whether young children’s early mathematical skills predicted future home numeracy practices.

In addition, many previous studies were conducted with western children, and far less was known with Chinese children. Several studies revealed that Chinese children showed better mathematics skills or numerical cognition than their western counterparts as early as preschool years (e.g., Siegler and Mu, 2008; Rodic et al., 2015), which may be partly attributed to family factors (Rodic et al., 2015). Compared with parents in western countries, Chinese parents place high expectations for their children’s school achievement, especially for mathematical achievement (Wang, 2004; Luo et al., 2013). In addition, Chinese parents of third-year kindergarten children start to rank academic skills as the most important area among children’s developmental outcomes (Chan, 2012; Lau, 2014), and may increase the frequency of academic activities during children’s transition to primary school. A handful of studies on Chinese young children showed the relationship between parent–child numeracy activities and early mathematical skills (Huang et al., 2017; Liu et al., 2019; Zhang et al., 2020). Most of them are concurrent design, and only one study (Zhang et al., 2020) showed that number application activities at the beginning of kindergarten predicted the increase in mathematics skills during the kindergarten year. However, to date, no studies examined how earlier early mathematical skills predicted the later home numeracy practices among Chinese young children.

### The present study

The aim of this study is to examine the mutual relationship between home numeracy practices (informal and formal numeracy activities) and early mathematics skills (number processing and arithmetic skills) among a group of Chinese young children. Random-interception cross-lagged panel modeling (RI-CLPM; Hamaker et al., 2015) is performed to examine their cross-lagged relationship. Compared with the traditional cross-lagged panel modeling, RI-CLPM examined the cross-lagged relationship between the within-personal individual difference of variables controlling for the between-personal individual difference (Hamaker et al., 2015). Since previous studies had not examined the effects of children’s mathematics skills on the home numeracy during the early years, we can only expect that the home numeracy at earlier time points may predict subsequent number processing and arithmetic skills based on the findings of previous studies (e.g., Vasilyeva et al., 2018; Susperreguy et al., 2020).

## Materials and methods

### Participants

A total of 155 Chinese children (82 boys, 73 girls; mean age = 67.49 months, SD = 3.58 months, range = 56–74 months) were recruited from one urban public kindergarten and two suburb public kindergartens in Shanghai, China (letters of information were initially sent to the parents of about 180 children). All three kindergartens are rated as level-one by the quality rating system at the city level.^1^ All children were attending the third year of kindergarten, and none were diagnosed with intellectual, sensory, or behavioral disorders. A total of 13 children withdrew from the study at the second or third wave because of their illness or moving to other kindergartens. The data were missing completely at random (MCAR) according to the results of Little’s MCAR test (χ^2^ = 77.2, df = 76, *p* = 0.44). Across three timepoints, about 60–70% of the questionnaires were completed by mothers, 30–40% by fathers, and less than 5% by grandparents. Most parents had three-year college studies or four-year university studies (63% fathers, 68% mothers), some of the parents had graduate studies (21% fathers, 14% mothers), and the remaining had high school studies or vocational school studies (8% fathers, 11% mothers), or primary or junior high school studies (8% fathers, 7% mothers).

### Materials

#### Home numeracy practices

Parents were asked to report how frequently they engaged in 13 numeracy-related activities (based on the original questionnaire of LeFevre et al., 2009; Huang et al., 2017) with their children in the recent month (e.g., ‘In the last month, how often did you work with your child on printing numbers?’) using a 5-point Likert scale (0 = never to 4 = almost daily). According to the structures in previous studies (e.g., LeFevre et al., 2009; Huang et al., 2017), three-factor models were constructed firstly: formal teaching was assessed with seven items (‘teaching counting’, ‘teaching skip counting’, ‘comparing size or magnitude’, ‘teaching compare or counting on computer’, ‘identifying numbers’, ‘printing numbers’, and ‘teaching simple arithmetic’), number games three items (‘playing card games’, ‘playing board games with dice or spinner’, and ‘playing computer games involving mathematics’), and number application three items (‘being timed’, ‘talking about money’, and ‘talking about stops on bus or subway’).

However, the three-factor model did not fit the data well, and the modification indices suggested that the residual errors of three items, ‘identifying numbers’, ‘printing numbers’, and ‘teaching simple arithmetic’, should be correlated. Therefore, the formal teaching variable was divided into two latent variables. Since three items were related to written numbers, the latent variable was named advanced teaching, and the left four items were named basic teaching. The modified four-factor model has acceptable or excellent fits to the data of three waves (T1: χ^2^ (59) = 86.380, *p* = 0.012, CFI = 0.953, TLI = 0.938, RMSEA = 0.054; T2: χ^2^ (59) = 102.364, *p* = 0.000, CFI = 0.929, TLI = 0.907, RMSEA = 0.071; T3: χ2 (59) = 77.560, *p* = 0.053, CFI = 0.962, TLI = 0.949, RMSEA = 0.048). Cronbach alpha for the four subscales at three timepoints ranged from 0.69 to 0.81.

#### Early mathematical skills

Digit Comparison from Nosworthy et al. (2013) was used to assess *Arabic number processing*. Children were presented with a booklet of 56 digit pairs (ranging from 1 to 9; e.g., 4|5, 6|8) and were asked to cross off the larger one as fast as possible in 1 min. The score in each task was the total corrects divided by the time. The Split-reliability in this study was 0.79, 0.74, and 0.84, respectively.

Numerical Operations from Wechsler Individual Achievement Test (WIAT-III; Wechsler, 2009) was used to assess *arithmetic skills*. A total of 60 items on addition, subtraction, multiplication, division, and more advanced arithmetic were arranged in increasing difficulty, and children were asked to write the answers to these items one by one. The test was discontinued after four consecutive errors. The score was the number of correct answers. Cronbach’s alpha reliability coefficient at three timepoints in this study was 0.86, 0.91, and 0.89, respectively.

#### Covariates

##### Children’s executive functions

Behavior Rating Inventory of Executive Function-Preschool version (BRIEF-P; Gioia et al., 2000) was used to assess children’s executive functions. In total 63 items on a three-point scale (1 = Never, 2 = Sometimes, and 3 = Often) assessed children’s difficulties in daily activities related to five components (inhibition, shift, emotional control, working memory, and plan/organize) of executive functions. The average score of all items, i.e., global executive composite score, was used. The Cronbach’s α reliability coefficient was 0.89 in this study.

##### Parent’s education levels

Parents were asked to report on their highest attained education on a 4-point scale ranging from 1 = finished elementary or secondary school to 4 = completed master’s or doctoral studies. The average of mother’s and father’s education scores was used.

### Procedure

Parental permission and ethical approval from the affiliation of the authors was obtained before testing. The participants were assessed three times every 4 months approximately, in November/October (T1, the beginning of the school year), February/March (T2), and May/June (T3). The parents completed the questionnaire including items on home numeracy practices, parents’ educational levels, and children’s executive functions, and the children were individually assessed in a quiet room at school by trained graduate students.

### Statistical analyses

RI-CLPM (Hamaker et al., 2015) was performed to examine the cross-lagged relationship between home numeracy practices and early mathematical skills. Separate models were constructed for formal numeracy practices (basic teaching activities, advanced teaching activities) and informal numeracy practices (number games, number application). In both models, children’s age, gender (0 = boy, 1 = girl), executive functions, parents’ education levels, and kindergarten (0 = urban, 1 = suburb) were added as covariates predicting the intercepts of home numeracy practices and early mathematical skills (Mulder and Hamaker, 2021). Both autoregressive and cross-lagged path estimates (e.g., basic teaching at T1 to Digit Comparison at T2, and basic teaching at T2 to Digit Comparison at T3) were constrained to be equal for each of home numeracy practices, Digit Comparison, and Numerical Operation. The constrained model was then compared to the nested model in which both autoregressive and cross-lagged parameters were freely estimated, and the more parsimonious model (i.e., the constrained model) would be used if the difference (chi-squared test) was non-significant (Bentler and Satorra, 2010). The RI-CLPM analysis was performed using the ‘lavaan’ package (Rosseel, 2012) for R software, and missing data were handled using Full Information Maximum Likelihood (FIML).

## Results

### Preliminary data analyses

Table 1 presents descriptive statistics (mean, standardized deviation, skewness, and kurtosis) for the measures of home numeracy practices and mathematics skills along with their Pearson correlations controlling for the covariates. According to the correlation matrix in Table 1, weak to moderate associates were found between four types of home numeracy activities and two early mathematical skills across three timepoints (*r*s ranged from −0.04 to 0.43). More specifically, formal teaching activities (basic and advanced teaching) weakly correlated with concurrent or later early mathematical skills (*r*s ranged from −0.04 to 0.28), and weak to moderate correlations were found between informal activities (number games and number application) and concurrent or later early mathematical skills (*r*s ranged from 0.11 to 0.43). Furthermore, earlier mathematics skills weakly correlated with later home numeracy activities (*r*s ranged from 0.02 to 0.27).

**Table 1:** Descriptive statistics for the measures of home numeracy and mathematics along with their correlations (controlling for the covariates).

Variable	M ± SD	Skew.	Kurt.	1	2	3	4	5	6	7	8	9	10	11	12	13	14	15	16	17
1. T1_BA	2.88 ± 0.92	−0.12	−0.72																	
2. T1_AA	3.59 ± 0.94	−0.39	−0.71	0.59**																
3. T1_NG	2.04 ± 0.90	0.92	0.37	0.24**	0.28**															
4. T1_NA	2.80 ± 0.84	0.16	−0.70	0.36**	0.34**	0.31**														
5. T1_DC	0.41 ± 0.09	0.04	−0.48	0.11	0.08	0.18*	0.19*													
6. T1_NO	15.14 ± 4.47	−0.73	1.28	0.23**	0.18	0.22*	0.18*	0.41**												
7. T2_BA	2.92 ± 0.96	0.20	−0.96	0.43**	0.39**	0.23**	0.45**	0.08	0.22*											
8. T2_AA	3.44 ± 1.08	−0.37	−0.66	0.31**	0.48**	0.32**	0.31**	0.14	0.21*	0.56**										
9. T2_NG	2.14 ± 0.84	0.74	−0.01	0.13	0.14	0.52**	0.29**	0.27**	0.26**	0.42**	0.32**									
10. T2_NA	2.82 ± 0.81	−0.12	−0.58	0.29**	0.29**	0.35**	0.40**	0.10	0.22**	0.52**	0.53**	0.40**								
11. T2_DC	0.46 ± 0.12	0.11	−0.21	0.19*	0.16*	0.30**	0.20*	0.70**	0.45**	0.11	0.18*	0.36**	0.12							
12. T2_NO	16.59 ± 4.25	0.04	1.78	0.23*	0.28**	0.43**	0.24**	0.44**	0.60**	0.17	0.20*	0.39**	0.24**	0.42**						
13. T3_BA	2.88 ± 0.89	−0.20	−0.82	0.40**	0.32**	0.11	0.41**	0.06	0.10	0.50**	0.38**	0.25**	0.37**	0.14	0.02					
14. T3_AA	3.49 ± 1.02	−0.27	−0.60	0.32**	0.28**	0.14	0.27**	0.15	0.05	0.47**	0.49**	0.18*	0.33**	0.14	0.02	0.57**				
15. T3_NG	2.26 ± 0.80	0.37	−0.83	0.22**	0.22**	0.38**	0.31**	0.12	0.10	0.29**	0.28**	0.54**	0.29**	0.16	0.18	0.32**	0.31**			
16. T3_NA	2.91 ± 0.80	−0.01	−0.34	0.28**	0.15	0.23*	0.43**	0.11	0.20*	0.43**	0.32**	0.26**	0.47**	0.12	0.09	0.57**	0.45**	0.42**		
17. T3_DC	0.52 ± 0.13	0.42	−0.11	0.09	0.13	0.21*	0.28**	0.57**	0.37**	0.19*	0.27**	0.23**	0.23**	0.66**	0.30**	0.22*	0.19*	0.11	0.21*	
18. T3_NO	17.96 ± 3.80	0.34	0.75	0.26**	0.18	0.34**	0.12	0.44**	0.64**	0.10	0.14	0.33**	0.23**	0.44**	0.77**	−0.03	−0.04	0.13	0.19*	0.25**

M, mean; SD, standard deviation; BA, basic teaching activities; AA, advanced teaching activities; NG, number games; NA, number application; DC, digit comparison; NO, numerical operation; covariates: gender (0, boy; 1, girl), children’s age, parents’ education levels, kindergarten (0, urban; 1, suburb).

* *p* < 0.05;

** *p* < 0.01.

### Results of random-interception cross-lagged panel modeling

**Figure 1:**
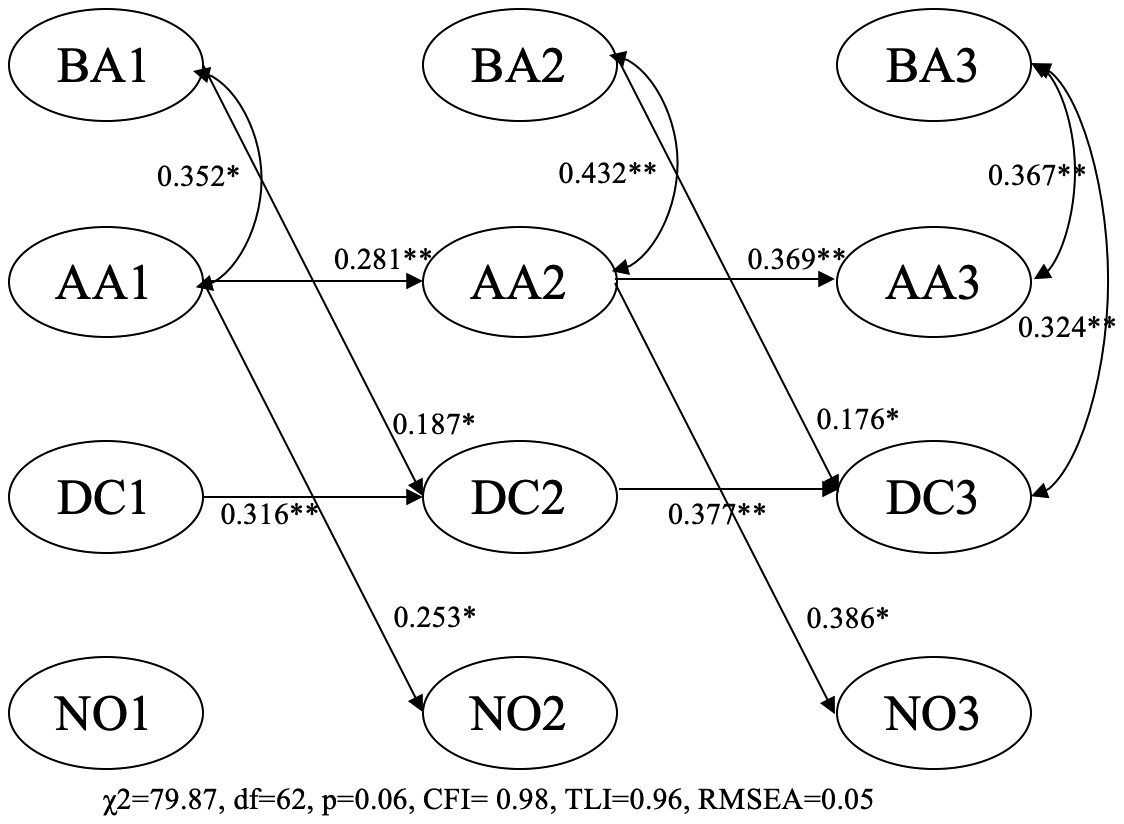
BA, basic teaching activities; AA, advanced teaching activities; DC, digit comparison; NO, numerical operation; children’s age, gender, executive functions and parents’ education were covariates predicting the intercepts of BA, AA, DC, and NO. *p < 0.05, **p < 0.01.

**Figure 2:**
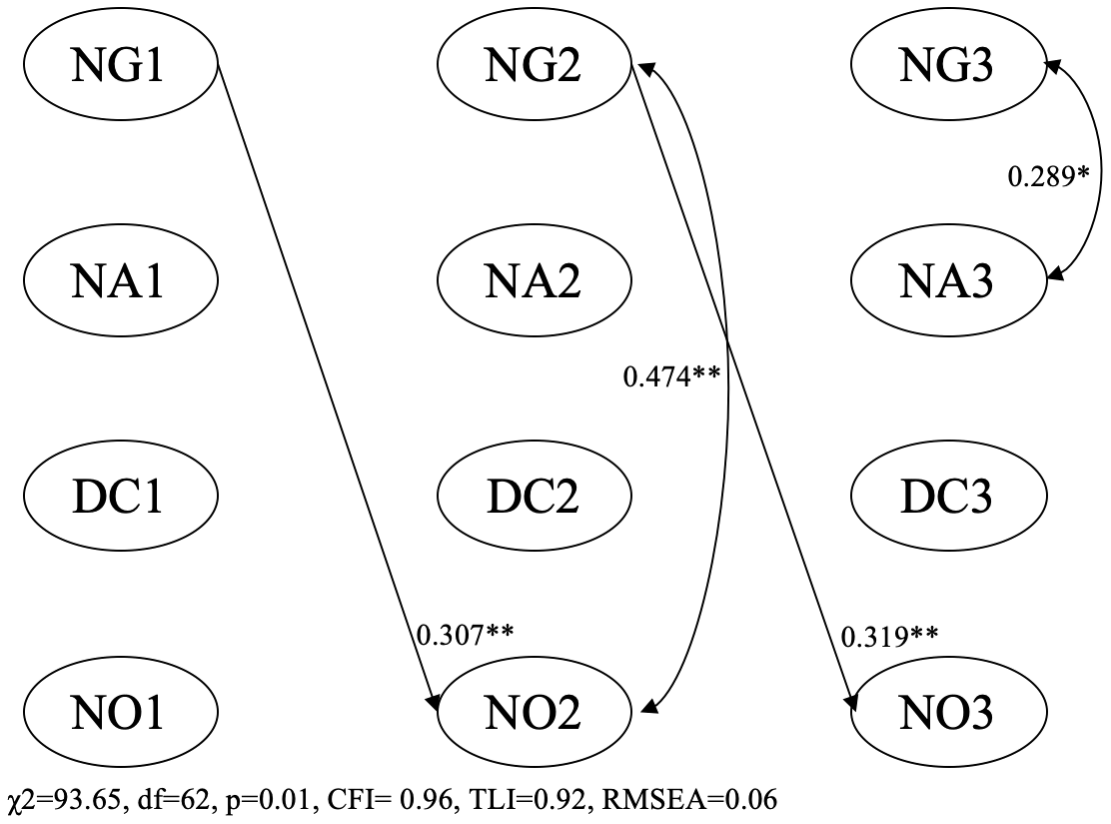
NG, number games activities; NA, number application activities; DC, digit comparison; NO, numerical operation; children’s age, gender, executive functions and parents’ education were covariates predicting the intercepts of NG, NA, DC, and NO. *p < 0.05, **p < 0.01.

The results of RI-CLPM were presented in Figures 1, 2, in which the intercepts and the covariates along with the non-significant paths were removed to simplify the models. Figure 1 showed the results for within-family relations among formal numeracy activities and mathematics skills. Insignificant difference was found between the constrained and free-estimated models (Δχ^2^ = 22.66, Δdf = 16, *p* = 0.12), and thus results of the constrained model were reported. The constrained model fitted the data well (χ^2^ = 79.87, df = 62, *p* = 0.06, CFI = 0.98, TLI = 0.96, RMSEA = 0.05). The estimates of the autoregressive parameters showed within-family associations over time for advanced teaching activities and Digit Comparison but not for basic teaching activities or Numerical Operation. It should be noted that the autoregressive effect in RI-CLPM (referred as carry-over effect in Hamaker et al., 2015) is different from that in traditional CLPM, since the rank-order stability of each variable across times in RI-CLPM is captured by the intercept of the variable (Hamaker et al., 2015). The estimates of the cross-lagged parameters showed that basic teaching activities significantly and positively predicted change in Digit Comparison, which in RI-CLPM implied that one child whose parents have more frequent basic teaching activities relative to their expected score (the means of the frequency of basic teaching activities across three timepoints), is likely to have higher performance on Digit Comparison relative to the child’s expected score at the next time point as well. The results also showed advanced teaching activities predicted change in Numerical Operation.

Figure 2 showed the results for within-family relations among informal numeracy activities and mathematics skills. Insignificant difference was also found between the constrained and free-estimated models (Δχ^2^ = 19.90, Δdf = 16, *p* = 0.22), and thus results of the constrained model were reported. The constrained model also fitted the data well (χ^2^ = 93.65, df = 62, *p* = 0.01, CFI = 0.96, TLI = 0.92, RMSEA = 0.06). The estimates of the cross-lagged parameters showed that number game activities significantly and positively predicted change in Numerical Operation.

## Discussion

This study aimed to examine the cross-lagged relationship between home numeracy practices and early mathematical skills during the kindergarten year in young Chinese children. The results showed that earlier basic teaching activities positively and significantly predicted the following number processing, and earlier advanced teaching activities along with earlier number games activities predicted subsequent arithmetic skills.

The different prediction power of basic and advanced teaching activities in early mathematic skills echoed the argument of Mutaf Yıldız et al., 2018. However, Mutaf Yıldız et al., 2018, in their review, claimed that advanced teaching activities instead of basic teaching activities predicted mathematics skills among four-to six-year-old children since they already had basic number knowledge. In comparison, earlier basic teaching activities uniquely predicted subsequent number processing in our study. The reason may be that number processing in our study assesses the efficiency of processing Arabic numbers instead of Arabic number knowledge, and children may also improve their number processing efficiency with repeated exposure to the magnitude in basic teaching activities.

Interestingly, number games in our study uniquely predicted arithmetic skills, which was in line with the findings of previous studies (Siegler and Ramani, 2009; Cheung and McBride, 2016). For example, Siegler and Ramani (2009) found that playing board games promoted low-income children’s performance on mathematics tasks (e.g., number identification, magnitude comparison, arithmetic). The study by Cheung and McBride (2016) showed that Chinese parents frequently used counting and addition in the number board game to demonstrate to their children, and thus may help children understand the combination of numbers.

Generally, the main results showed that earlier home numeracy practices unidirectionally predicted subsequent early mathematics skills, and did not replicate the effects of children’s mathematics on home numeracy practices in previous studies on primary students (e.g., Silinskas et al., 2010, 2020; Deng et al., 2015). Our results thus did not support the responsive model of home numeracy practices (Silinskas et al., 2020) during the early years. Considering Chinese parents’ high expectations for children’s academic performance (Luo et al., 2013), the reason cannot be that parents are not sensitive to their children’s mathematics skills. One possible reason may be that parents would get less explicit reports of children’s mathematics from the kindergarten teachers, and their perceptions of children’s mathematics skills may be imprecise. Parents of primary students can learn about the mathematics performance of their children through report cards or homework (Núñez et al., 2017), and thus may provide more frequent numeracy activities to facilitate children’s mathematics learning. However, these explicit feedbacks are typically unavailable during the kindergarten year, and thus parents’ perception of young children’s actual mathematics skills may be imprecise. Some studies compared the mathematics skills reported by parents and the objective performance of children on early mathematics tests, and found their correlation was very low (e.g., Sonnenschein et al., 2014). Moreover, it could be worse under the low frequency of family-kindergarten communication (Rimm-Kaufman and Pianta, 1999). As a result, parents may misestimate their children’s mathematics performance (Pezdek et al., 2002), and thus may not provide appropriate numeracy activities scaffolding children’s early mathematics learning.

Some limitations should be mentioned. First, the frequency of home numeracy practices was reported by parents in this study, and thus the results may be biased by parents’ social desirability (Elliott and Bachman, 2018). Further studies may use more objective measurements such as direct observation or activity checklist. Second, children in our study attended the third year of kindergarten (5–6 years old), and thus the findings may not be generalized to younger preschool children (2–4 years old). Finally, the sample size of this study was relatively small for running RI-CLPM analysis despite the well-fitting results, and future studies may recruit and examine more participants.

Despite these limitations, the strength of this study is using a three-wave longitudinal study examining the bidirectional relationship between home numeracy practices and children’s early mathematics skills. The first implication is that parents may provide both formal and informal numeracy activities to promote children’s early mathematics development, considering the predictive power of both types of home numeracy practices. The second implication is that parents may observe and monitor their children’s mathematics progress through more reliable approaches, such as collaborating with kindergarten teachers.

## Data availability statement

The raw data and the R scripts supporting the conclusions of this article are available from the first author, WW (wwei@shnu.edu.cn), upon reasonable request.

## Ethics statement

The studies involving human participants were reviewed and approved by Shanghai Normal University Ethics Committee. Written informed consent to participate in this study was provided by the participants’ legal guardian/next of kin.

## Author contributions

WW and YL: research design. WW, Q-YW, and QL: data collection. WW, Q-YW, and YL: data analysis. All authors contributed to the article and approved the submitted version.

## Funding

This research was funded by the Ministry of Education in China Project of Humanities and Social Sciences (No. 18YJC880086).

## Conflict of interest

The authors declare that the research was conducted in the absence of any commercial or financial relationships that could be construed as a potential conflict of interest.

## Publisher’s note

All claims expressed in this article are solely those of the authors and do not necessarily represent those of their affiliated organizations, or those of the publisher, the editors and the reviewers. Any product that may be evaluated in this article, or claim that may be made by its manufacturer, is not guaranteed or endorsed by the publisher.

## Footnotes

^1^Kindergartens in Shanghai are qualified into five levels, i.e., city-level demonstrative, district-level demonstrative, level-one, level-two, and level-three, and more than half are rated as level-one.
